# Enterohaemorrhagic *E. coli* modulates an ARF6:Rab35 signaling axis to prevent recycling endosome maturation during infection

**DOI:** 10.1016/j.jmb.2016.05.023

**Published:** 2016-08-28

**Authors:** R. Christopher D. Furniss, Sabrina Slater, Gad Frankel, Abigail Clements

**Affiliations:** MRC Centre for Molecular Bacteriology and Infection, Department of Life Sciences, Imperial College London, London, SW7 2AZ

**Keywords:** ARF, ADP ribosylation factor, T3SS, Type III secretion system, PM, plasma membrane, RE, recycling endosome, TfR, transferrin receptor, GFP, green fluorescent protein, A/E, attaching and effacing, *E. coli*, *Escherichia coli*, OCRL, oculocerebrorenal syndrome of Lowe, CCV, clathrin coated vesicles, EEA1, early endosome antigen 1, CLC, clathrin light chain, Type 3 secretion system, EspG, host-pathogen interactions, small GTPase signaling, endosomal recycling, cargo trafficking

## Abstract

Enteropathogenic and enterohaemorrhagic *Escherichia coli* (EPEC/EHEC) manipulate a plethora of host cell processes to establish infection of the gut mucosa. This manipulation is achieved via the injection of bacterial effector proteins into host cells using a Type III secretion system. We have previously reported that the conserved EHEC and EPEC effector EspG disrupts recycling endosome function, reducing cell surface levels of host receptors through accumulation of recycling cargo within the host cell. Here we report that EspG interacts specifically with the small GTPases ARF6 and Rab35 during infection. These interactions target EspG to endosomes and prevent Rab35-mediated recycling of cargo to the host cell surface. Furthermore, we show that EspG has no effect on Rab35-mediated uncoating of newly formed endosomes, and instead leads to the formation of enlarged EspG/TfR/Rab11 positive, EEA1/Clathrin negative stalled recycling structures. Thus, this paper provides a molecular framework to explain how EspG disrupts recycling whilst also reporting the first known simultaneous targeting of ARF6 and Rab35 by a bacterial pathogen.

## Highlights

• EHEC delivers effector proteins into host cells to establish infection in the gut
• The effector EspG interacts with GTP-ARF6 confining EspG to recycling endosomes
• During infection EspG interacts preferentially with Rab35, not Rab1
• Spatial restriction of bacterial effectors during infection determines their function

## Graphical abstract

**Figure:**
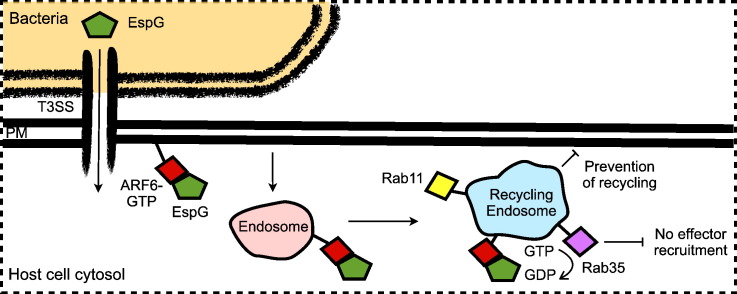

## Introduction

The attaching and effacing (A/E) pathogens enterohaemorrhagic *Escherichia coli* (EHEC) and enteropathogenic *E. coli* (EPEC) use a type III secretion system (T3SS) to deliver an array of bacterial effector proteins into host cells during infection, facilitating colonization of the gut epithelia [1]. EHEC regularly causes food-poisoning outbreaks, with associated diarrhea, hemorrhagic colitis and hemolytic uremic syndrome, and remains the leading cause of acute pediatric renal failure in the UK and US [2], [3]. EPEC causes illness in young children in low-income countries and is responsible for significant morbidity and mortality due to diarrheal disease.

Recently we demonstrated that EHEC depletes a number of cell surface receptors from the Plasma Membrane (PM) during infection, in a manner dependent on the T3SS effector EspG [4]. Amongst the cell surface receptors depleted is the Transferrin Receptor (TfR), the prototypical recycling protein, which cycles between the PM and the early and recycling endosome compartments [5]. We demonstrated that the reduction in TfR levels on the cell surface is not due to degradation, as total cellular levels of TfR are unaltered during infection, and that injection of EspG results in the movement of the TfR to cytosolic vesicles positive for markers of recycling endosomes [4], [6], [7]. Therefore, we hypothesize that EspG may inhibit the recycling of internalized cell surface receptors back to the PM. Glotfelty et al. (2014) [8] recently described a similar observation, reporting the accumulation of internalized occludin within EPEC infected cells and an EspG1/G2-dependent accumulation of other tight junction proteins within the cytosol. Gill et al. (2007) [9] described the EspG-dependent movement of the major apical anion exchanger DRA away from the PM to intracellular compartments during EPEC infection and showed that EspG-mediated reduction of DRA at the plasma membrane is due to a decrease in DRA exocytosis [10]. These observations support our hypothesis that EspG influences the protein composition of the host plasma membrane through modulation of recycling endosomes.

Since its discovery in 2001 [11] the role of EspG has been extensively studied [9], [12], [13], [14], [15], [16], [17], [18], [19], although its function during infection has remained controversial. EspG is now understood to modulate host endomembrane trafficking by functioning as a “catalytic scaffold” [20]. EspG has been shown to bind active, GTP-bound, ARF GTPases and to act as a Rab GTPase activating protein (GAP) [21]. Co-crystallization experiments [21] suggest EspG can bind ARF6 and Rab1 on its opposing surfaces, in a similar manner to the interactions originally described between EspG, ARF1 and p21-activated kinase (PAK) [20], raising the possibility that EspG may reorganize multiple host signaling networks during infection.

Our investigation of the small GTPase interacting partners of EspG during infection reveals that EspG modulates an ARF6:Rab35 signaling axis to disrupt recycling endosome function, resulting in the accumulation of recycling cargo within the host cytosol. Our results highlight the importance of spatial restriction of bacterial effector proteins during infection, whilst simultaneously providing a molecular mechanism to support previously published EspG phenotypes.

## EspG interacts with ARF6 during infection

**Fig. 1:**
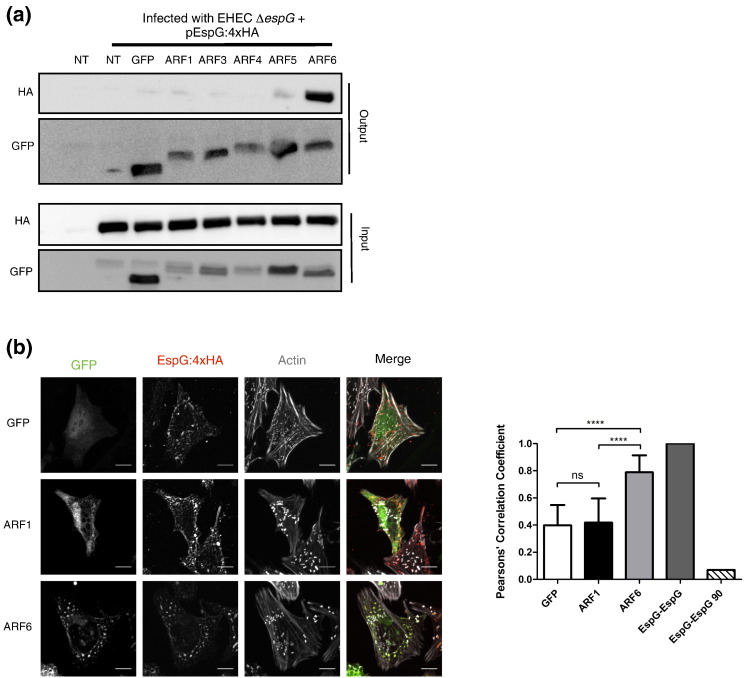
**EspG interacts with ARF6 during infection**. a) HeLa cells expressing GFP-ARF fusions were infected with EHEC Δ*espG* + pEspG:4xHA. After 5 h of infection ARFs were immunoprecipitated (output, GFP) and co-immunoprecipitated EspG:4xHA detected (output, HA) (n = 4, see Fig. S1 for additional blots) b) Confocal microscopy suggests that GFP-ARF6 and EspG:4xHA colocalize on the same endosomal structures. Representative images show maximum intensity Z-projections, scale bars represent 5 μm. Colocalization was quantified using Pearsons' Correlation Coefficients generated for 20 fields of view (1–2 cells per image) across 2 independent experiments, graph shows means ± SD, ns = non-significant, **** = p < 0.0001.

Humans express multiple ARF [5] and Rab (60 +) proteins, each involved in specific membrane trafficking events (e.g. endoplasmic reticulum to Golgi, or early to late endosome). *In vitro* data indicates EspG can interact with at least 3 of the 5 ARF proteins (ARF 1, 5 and 6) [20]. However, the specificity of EspG interactions during infection remains unclear. To determine which ARF GTPases are genuine EspG interacting partners during infection we performed co-immunoprecipitation experiments using HeLa cells expressing a panel of GFP-tagged ARF GTPases infected with EHEC ∆*espG* + pEspG:4xHA (Fig. 1, replicate blots shown in Fig. S1). This revealed that EspG interacts primarily with ARF6 (Fig. 1a). ARF6 was also found to be the primary interacting partner over an infection time-course from 2.5 to 7.5 h of infection (data not shown), suggesting the ARF6 interaction occurs early during infection and is maintained as infection progresses.

Our previous observations regarding the localization of EspG during infection suggest EspG is not trafficking to the Golgi, as seen during ectopic expression [18], [21] but is instead localized in endosomal compartments, with markers of recycling endosomes [4]. ARF 1,3, 4 and 5 have previously been described to localize predominantly to the Golgi [22] while ARF6 is mainly found at the plasma membrane and at endosomal sites [23]. These localizations were confirmed for the GFP-ARF fusions used in this work (Fig. S2). We observed that during EHEC infection ARF6 and EspG accumulated on the same endosomal structures. Calculation of Pearsons' Correlation Coefficients for cells transfected with GFP-ARF6 and infected with EHEC ∆*espG* + pEspG:4xHA indicates specific co-localization between ARF6 and EspG (Fig. 1b) consistent with the co-immunoprecipitation data (Fig. 1a).

## EspG preferentially interacts with active ARF6 to localize at endosomal structures during infection

**Fig. 2:**
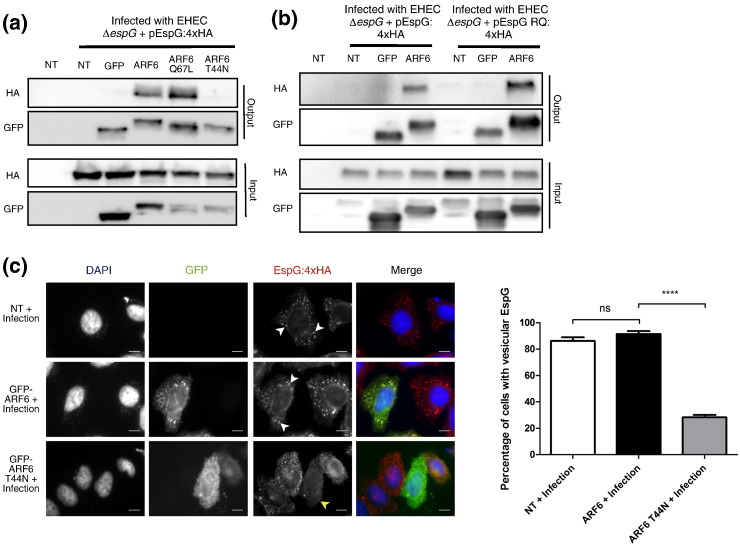
**EspG preferentially interacts with GTP-ARF6 upstream of Rab binding to target itself to endosomal structures.** a) HeLa cells expressing GFP-ARF6 Q67L (GTP-locked) and T44N (GDP-locked) were infected with EHEC Δ*espG* + pEspG:4xHA. After 5 h of infection ARFs were immunoprecipitated (output, GFP) and co-immunoprecipitated EspG:4xHA detected (output, HA) (n = 4) b) HeLa cells expressing GFP-ARF6 were then infected with EHEC Δ*espG* + pEspG:4xHA or EHEC Δ*espG* + pEspG RQ:4xHA, GFP-ARF6 immunoprecipitated and co-immunoprecipitated EspG:4xHA or EspG RQ:4xHA detected (n = 2). c). HeLa cells expressing GFP-ARF6 or GFP-ARF6 T44N were infected with EHEC ∆*espG* + pEspG:4xHA for 5 h before the percentage of transfected cells exhibiting endosomal EspG:4xHA staining was scored. White arrowheads indicate EspG positive endosomal structures; yellow arrowheads indicate diffuse cytosolic EspG staining in cells expressing GFP-ARF6 T44N. Scale bars represent 10 μm. Graph shows means ± SD of three independent experiments (> 100 cells counted per experiment) ns = non-significant, **** = p < 0.0001.

To determine if EspG interacts preferentially with GTP-bound ARF6 during infection we assessed the co-immunoprecipitation of EspG by constitutively inactive, GDP-bound (T44N) or constitutively active, GTP-bound (Q67L) ARF6 mutants [24]. Consistent with previous *in vitro* data [25], EspG was preferentially co-immunoprecipitated with GTP-bound rather than GDP-bound ARF6 (Fig. 2a).

ARF-binding has been proposed to spatially restrict EspG within host cells to allowing targeted, local Rab inactivation [21], [25]. We therefore reasoned that ARF6 binding occurs upstream of interaction with Rabs and should therefore be independent of EspG's ability to act as a Rab GAP. Consistent with this hypothesis, an EspG Rab GAP mutant (EspG RQ) was co-immunoprecipitated by ARF6 during EHEC infection as efficiently as WT EspG (Fig. 2b). This hypothesis was further confirmed by the observation that cells expressing GDP-locked GFP-ARF6 T44N fail to show the characteristic endosomal localization of EspG, in contrast to un-transfected cells or those expressing WT GFP-ARF6 (Fig. 2c). Therefore the interaction of EspG with GTP-ARF6 is required for correct localization of EspG and is independent of EspG's Rab GAP activity.

## EspG interacts with Rab35 during infection

Over 60 human Rab isoforms have been described to date [26]. We screened a panel of Rabs that have been described to localize at recycling endosomes or the trans-Golgi network [27]. This panel consisted of Rabs 8, 10, 11, 13, 22a, 30, 35, 37, 38 and 43a (Fig. S3). *In vitro* EspG was shown to act as a Rab GAP for only 12 of the 30 Rabs tested, including, in our panel, Rab 13, 30, 35, 37 and 38 [21]. We also included Rab1 in our panel as, *in vitro*, EspG showed the highest GAP activity for this protein [21].

**Fig. 3:**
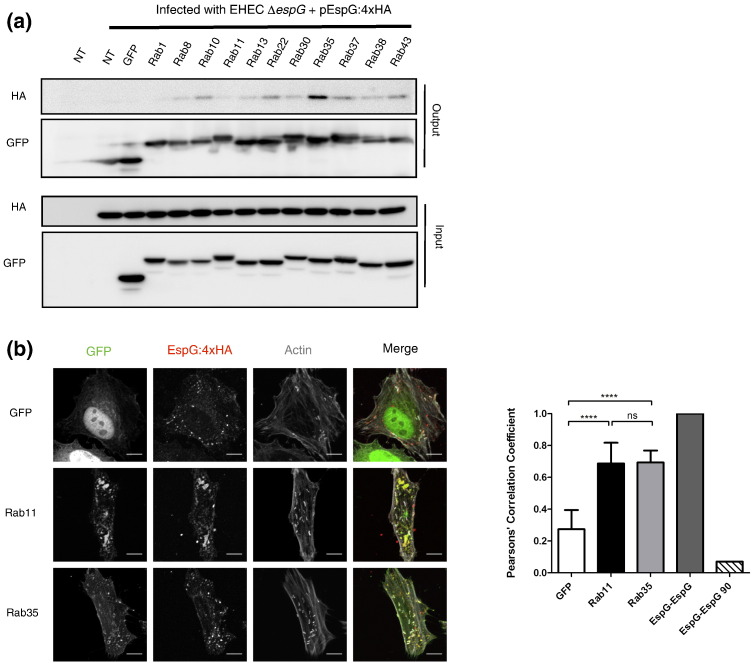
**EspG interacts and colocalizes with Rab35 during infection** a) HeLa cells expressing GFP-Rab fusions were infected with EHEC Δ*espG* + pEspG:4xHA. After 5 h of infection Rabs were immunoprecipitated (output, GFP) and co-immunoprecipitated EspG:4xHA detected (output, HA) (n = 5 for all Rabs except Rab8 where n = 2, see Fig. S4 for additional blots) b) Confocal microscopy suggests that both GFP-Rab11 and GFP-Rab35 colocalize with EspG:4xHA on the same endosomal structures. Representative images show maximum intensity Z-projections, scale bars represent 5 μm. Colocalization was quantified using Pearson's Correlation Coefficients generated for 20 fields of view (1–2 cells per image) across 2 independent experiments, graph shows means ± SD, ns = non-significant, **** = p < 0.0001.

Co-immunoprecipitation experiments using our Rab GTPase panel show that EspG is selectively and consistently immunoprecipitated by Rab35 (Fig. 3a, replicate blots shown in Fig. S4). EspG was also intermittently immunoprecipitated by Rab13, and then in decreasing amounts by Rabs 43a, 37 and 10. However Rab35 was the only Rab to immunoprecipitate EspG in every experiment. EspG was not co-immunoprecipitated by Rab1 in any experiment, suggesting it is spatially removed from EspG during infection. Interestingly, Rab1 and Rab35 share significant sequence similarity at the amino acid level, and cluster in a distinct subfamily of Rab GTPases [28]. Therefore, whilst EspG is able to interact with and induce GTP hydrolysis of both Rab1 and Rab35 *in vitro*, spatial restriction of EspG to endosomal compartments during infection appears to direct EspG's GAP activity towards Rab35.

Co-localization analysis of EspG and GFP-Rab constructs shows that EspG co-localizes with Rab35, supporting our co-immunoprecipitation data (Fig. 3b). EspG also co-localizes with Rab11, a known marker of recycling endosomes. However, co-immunoprecipitation of EspG by Rab11 was found to be no higher than with GFP alone, consistent with the absence of Rab GAP activity towards Rab11 *in vitro* [21]. These results indicate EspG localizes to ARF6/Rab35/Rab11 positive recycling endosomes during infection.

We propose that whilst EspG is capable of interacting with multiple ARF and Rab GTPases *in vitro*, during infection the spatial restriction of EspG limits its interacting partners. As such, it is the interactions with ARF6 (Fig. 1a) and Rab35 (Fig. 3a) that are relevant for EspG's function during infection. Our data suggests that during infection EspG is recruited to ARF6/Rab35 positive endosomal structures (Figs. 1c and 3c) through scaffolding with GTP-bound ARF6 (Figs. 1a and 2a). Targeting of EspG to these endosomal structures, via ARF6 (Fig. 2c), is necessary to ensure Rab hydrolysis only occurs for a specific Rab population, as previously postulated by Selyunin et al. [25]. During infection, this results in the Rab GAP activity of EspG being directed towards Rab35 (Fig. 3a).

Selyunin *et al.* also observed that binding of EspG to ARF-GTP may prevent access by ARF GAPs, thus locking the EspG-bound ARF in the GTP bound state. In this conformation the effector-binding surface of the ARF is unobstructed [25] raising the possibility that EspG, aside from using ARF-binding as a method of spatial restriction, also promotes the recruitment of ARF6 effectors. Importantly, as Rab GAP deficient EspG is able to interact with ARF6 (Fig. 2b), but unable to disrupt recycling [4], the functional disruption of REs during infection appears to be dependent on EspG's Rab GAP activity. This does not preclude the stabilization of active ARF6 by EspG from playing another, currently unknown, role during infection.

## Modulation of Rab35 by EspG does not influence vesicle uncoating after Clathrin-dependent endocytosis

**Fig. 4:**
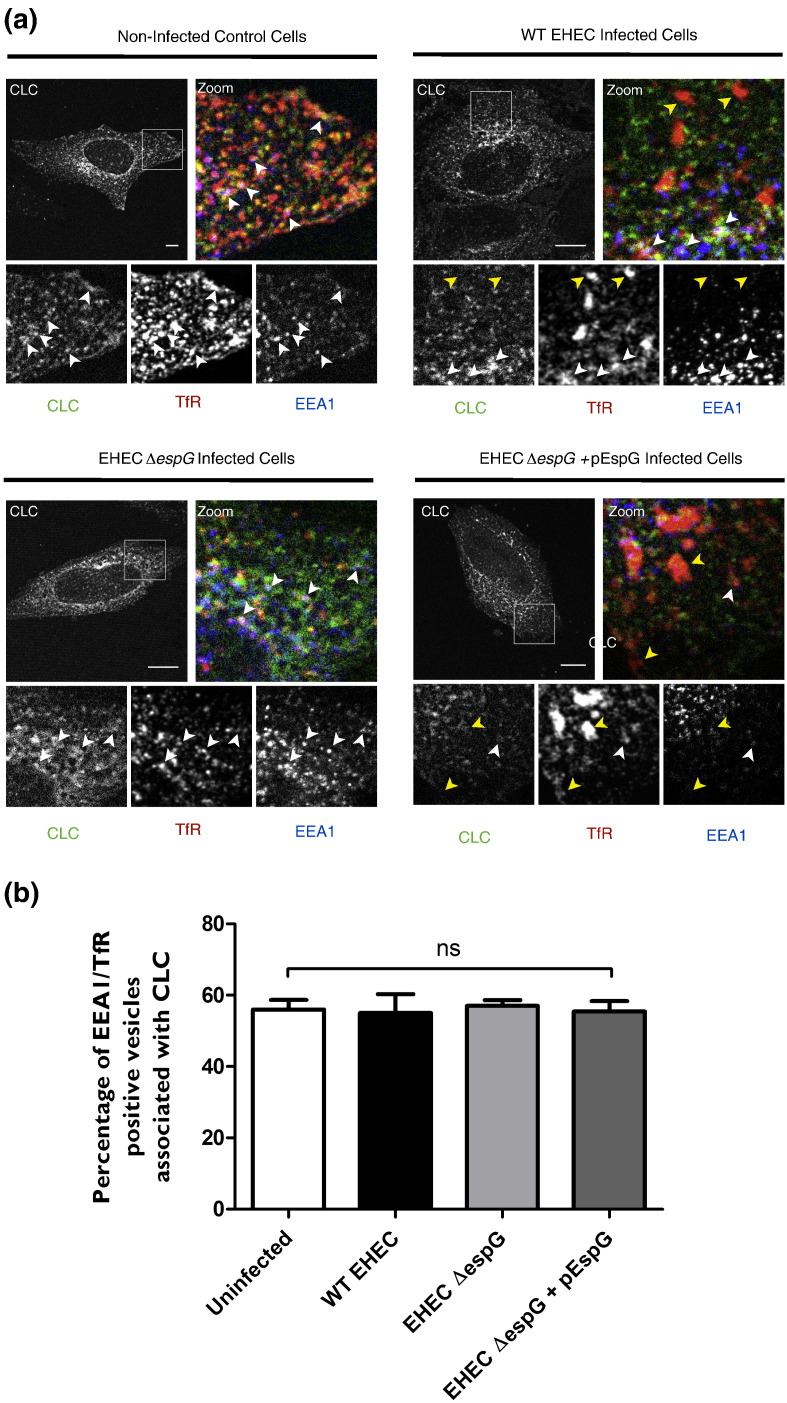
**EspG has no effect on the uncoating of TfR/EEA1 positive vesicles** A) HeLa cells expressing mRFP-CLC were infected with EHEC WT, a Δ*espG* mutant or Δ*espG* + pEspG:4xHA. After 5 h of infection cells were fixed and labeled for EEA1 and TfR. During WT EHEC infection, enlarged TfR positive, EEA1/CLC negative structures can be seen in infected cells. These structures were not observed in non-infected cells, nor those infected with EHEC ∆*espG*, but were more pronounced in cells infected with EHEC Δ*espG* + pEspG:4xHA. Scale bars represent 10 μm, white arrow heads indicate triple-positive structures, yellow arrow heads indicate large TfR-positive structures lacking EEA1/CLC. B) Quantification of images shows that EspG has no effect on the association of clathrin with TfR positive early endosomes (n = 2, > 150 EEA1/TfR positive structures scored per condition per experiment, graph is representative of one independent experiment, ns = non-significant).

Rab35 has been implicated in both endocytosis [29] and recycling of cargo [30], [31], [32]. We have previously demonstrated that EspG is able to disrupt cargo recycling [4] and were interested in whether EspG could also affect other Rab35 dependent activities. A recent report indicates that Rab35 has a role in the recruitment of Oculocerebrorenal Syndrome of Lowe (OCRL), an Inositol Polyphosphate 5-Phosphatase, to newly formed endosomes. Recruitment of OCRL promotes the uncoating of Clathrin-Coated Vesicles (CCVs) after endocytosis [29]. To assess the effect of EspG on clathrin-uncoating of vesicles, HeLa cells expressing mRFP-clathrin light chain (CLC) were infected with WT EHEC, EHEC ∆*espG* and EHEC ∆*espG* + pEspG:4xHA and stained for TfR and Early Endosome Antigen 1 (EEA1). Peripheral endosomes containing TfR and EEA1 showed similar association with CLC in all conditions (Fig. 4a and b), unlike in Rab35 and OCRL siRNA treated cells [29]. However large TfR positive structures could be observed in WT EHEC, and to a greater extent in EHEC ∆*espG* + pEspG:4xHA infected cells, but not in uninfected cells or cells infected with EHEC ∆*espG.* These large TfR positive structures were negative for both EEA1 and CLC (Fig. 4b), and resemble the enlarged EspG/TfR positive vesicular structures seen previously [4]. These data suggest that EspG specifically targets Rab35 involved in recycling and not in recruitment of OCRL and CCV uncoating after endocytosis.

Whilst Rab35 has been implicated in early endocytic processes [29], [33] ARF6 and Rab35 are also known to act antagonistically to control RE function, with ARF6 promoting the internalization of cell surface proteins and Rab35 mediating their recycling [30] via the assembly of a RE-bound signaling complex [32]. This Rab35-controlled complex also influences RE lipid composition [34] and vesiculation [31], [35]. EspG appears to have no effect on CCV uncoating, whilst large TfR positive but EEA1 and Clathrin negative structures occur in the presence of EspG. We therefore hypothesize that EspG potentiates its effects via the modulation of Rab35 specifically on recycling endosomes. Inactivation of Rab35 involved in recycling would give rise to the reduced cell surface proteins we [4], and others [8], [10] have previously observed during EHEC/EPEC infection. Consistent with this hypothesis, it was recently reported that knock-down of Rab35 in T_H_2 cells interferes with TfR recycling [36]. The RE vesiculation regulators EHD1 and GRAF1 form a complex with MICAL-L1 to promote budding of REs back to the PM [31], [35]. As MICAL-L1 can be recruited to REs by Rab35 [32], the inactivation of Rab35 by EspG may prevent RE vesiculation. This closely matches the phenotype observed when Rab35 function is perturbed by other means [37], but further work is required to fully uncover the details of EspGs effect on RE vesiculation.

Whilst a number of reports in the literature have identified ARF6 and Rab35 individually as targets during bacterial infection (ARF6 is implicated in host cell invasion by both *Shigella flexneri* [38] and *Salmonella enterica* serovar Typhimurium [39] and Rab35 is known to be modulated by both uropathogenic *E. coli* (UPEC) [40] and the *Legionella pneumophilia* Dot/Icm effector AnkX [41]) concurrent modulation of the ARF6:Rab35 signaling axis by EHEC EspG represents a previously unappreciated strategy of host cell modulation by a bacterial pathogen. As such, this work not only increases our understanding of the plethora of mechanisms bacterial pathogens use to subvert host cell functions but will also contribute to our understanding of the wide variety of fundamental host cell processes that ARF6 and Rab35 have been implicated in, including the AKT signaling pathway [42], sorting of newly endocytosed cargo from the plasma membrane [43], cytokinesis [44] and the establishment of epithelial cell polarity [45].

## Appendix A Supplementary data and methods

Supplementary material Image 2
