# Supplementary material for: Enterohaemorrhagic E. coli modulates an ARF6:Rab35 signaling axis to prevent recycling endosome maturation during infection
Source: J Mol Biol. 2016 Aug 28;428(17):3399–407. doi: 10.1016/j.jmb.2016.05.023 (PMC5013874; doi:10.1016/j.jmb.2016.05.023)
Supplement: Supplementary file 1 — Supplementary material [file mmc1.pdf]

Supplementary Figure 1

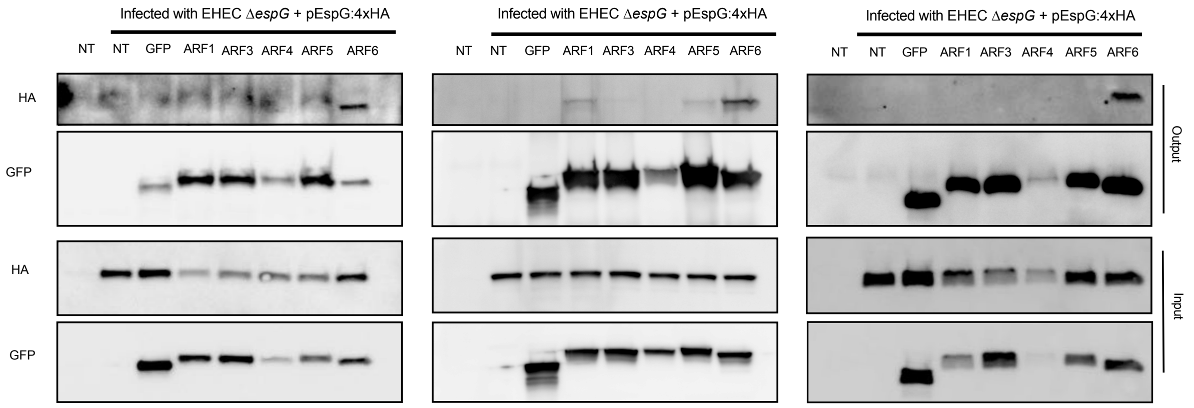

Figure S1: **GFP-ARF co-immunoprecipitation western blots**

HeLa cells expressing GFP-ARF fusions were infected with EDL933 Δ*espG* + pEspG:4xHA, GFP-Rabs were immunoprecipitated (output, GFP) after 5 hours of infection and co-immunoprecipitated EspG:4xHA detected (output, HA), as described in the main text. All blots, extra to those shown in Fig. 1, are presented here.

Supplementary Figure 2

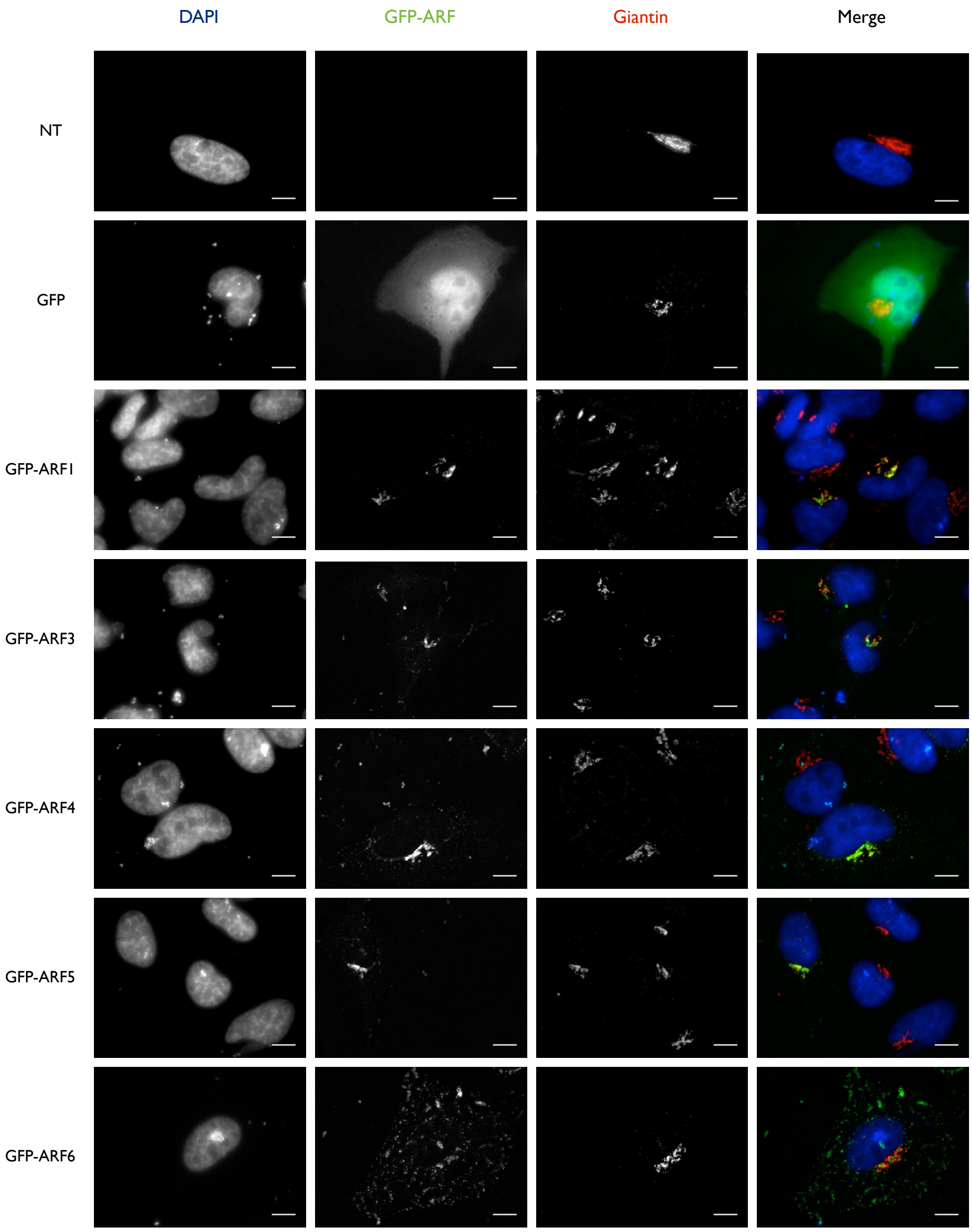

Figure S2: **GFP-ARF construct localization during transfection**

HeLa cells transfected for 18-24 hours with GFP-ARF fusions were fixed and stained for DAPI and the Golgi maker Giantin. GFP-ARF1, 3, 4 and 5 can be seen associated with the Golgi, as expected, whilst GFP-ARF6 can be seen

at endosomal structures. Representative images show maximum intensity Z-projections, scale bars represent 10 µm.

Supplementary Figure 3

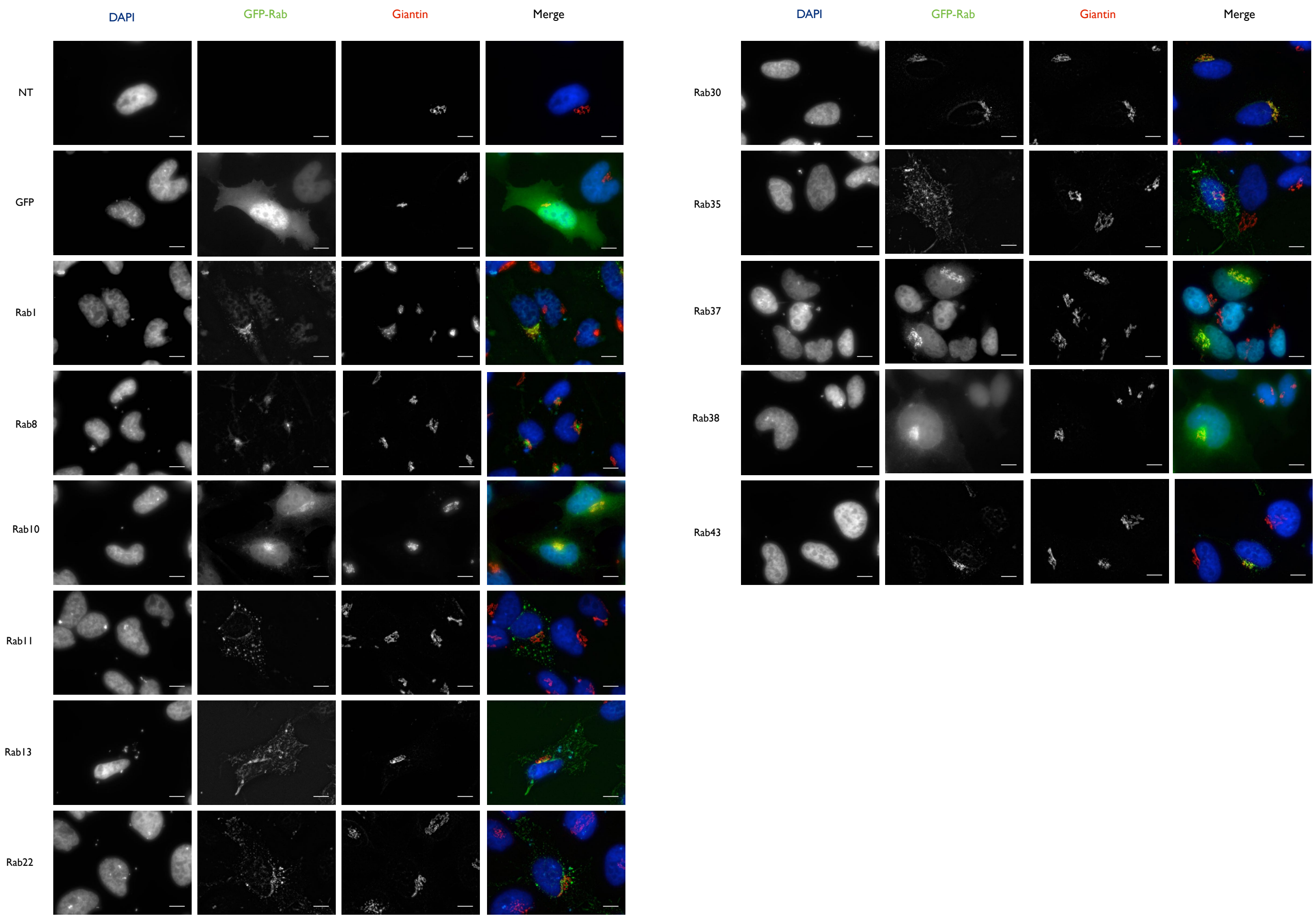

Figure S3: **GFP-Rab construct localization during transfection**

HeLa cells transfected for 18-24 hours with GFP-Rab fusions were fixed and stained for DAPI and the Golgi maker Giantin. All GFP-Rab fusions can be seen at the expected sub-cellular locations. Representative images show maximum intensity Z-projections, scale bars represent 10 µm.

Supplementary Figure 4

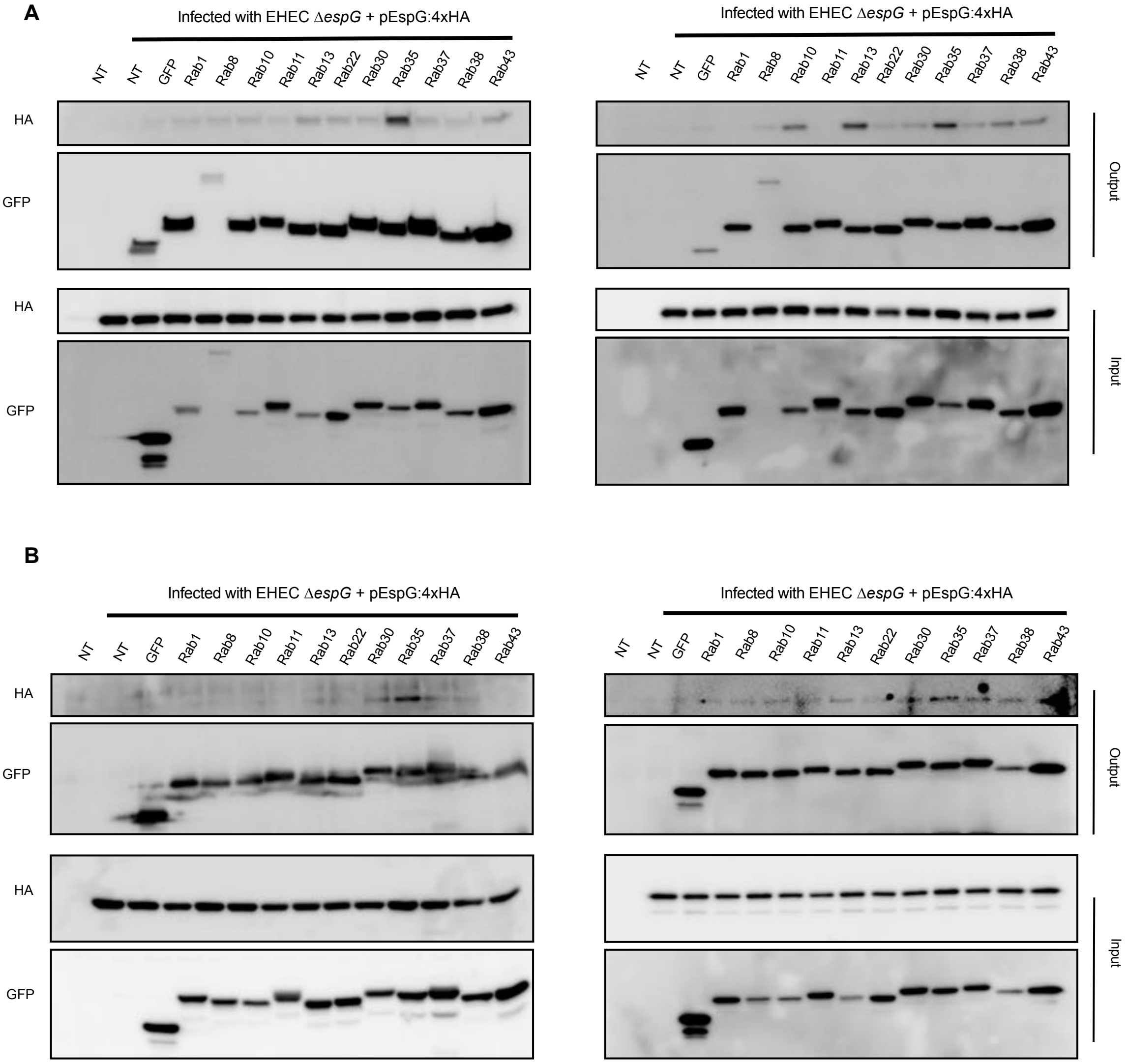

Figure S4: **GFP-Rab co-immunoprecipitation western blots**

HeLa cells expressing GFP-Rab fusions were infected with EDL933 Δ*espG* + pEspG:4xHA, GFP-Rabs were immunoprecipitated (output, GFP) after 5 hours of infection and co-immunoprecipitated EspG:4xHA detected (output, HA), as described in the main text. A) Western blots showing all GFP-Rab constructs, bar Rab8. B) Western blots showing all GFP-Rab constructs. All blots, extra to those shown in Fig. 3, are presented here.

Experimental Procedures

Bacterial Strains, Cell Culture and Infections

espG was deleted from EHEC strain EDL933 and pSA10 plasmids containing EspG and EspG RQ with C-terminal 4xHA fusions were created as previously described[1]. HeLa cells (ATCC) were maintained in DMEM (1 g/L glucose, 2 mM glutamax and 10% (v/v) FCS) and seeded at $7.4x10^4$ cells/well in 24 well plates with coverslips for microscopy or $2.8x10^5$ cells/well in 6 well plates for co-IP, 48h prior to infection. For infection experiments, *E. coli* EDL933 strains were routinely grown in Luria Bertani (LB) medium (Merck) for 8 hours at 37°C with aeration and appropriate antibiotic selection, before subculture in DMEM (1 g/L glucose) (Sigma Aldrich) for 16-18 hours, 37°C 5% CO2. When required 0.2 mM IPTG was added to cultures 30 minutes prior to infection. EHEC cultures were diluted in DMEM (1 g/L glucose) and added to cells at an MOI of approximately 100:1. Plates were centrifuged at 500 *x g* for 5 minutes to synchronize infection. Infected cells were incubated for 2.5 hours before washing 3 x PBS and addition of DMEM (1 g/L glucose + 100 µg/mL gentamicin). As required, cells were transfected 24 hours prior to infection using Genejuice (Merck) according to the manufacturers instructions. Constructs were obtained from the following sources: peGFPN1-ARF1,3,4 and 5 (Chun *et al.* 2008[2],Addgene 39554-7), peGFPN1-ARF6 WT, Q67L, T44N and pmRFP-CLC[3] were a kind gift from Philippe Chavrier (Institut Curie, Paris), pCS2-GFP-Rab 1a, 8,10, 11, 13, 22, 30, 35, 37 and 43a were a kind gift from Feng Shao (National Institute of Biological Sciences, Beijing).

Immunofluorescence Microscopy

Coverslips for analysis were washed 3 x PBS and fixed with 2% paraformaldehyde (PFA)/PBS for 20 minutes at RT, before addition of 10 mM NH_4Cl. Cells were permeabilized for 8 minutes with 0.1% Triton X-100 prior to blocking with 3% BSA/PBS for 30 minutes. DNA was stained with DAPI (1:1000, Invitrogen) EspG:4xHA was detected using rabbit anti-HA.11, clone 16B12 primary antibody (1:1000, Cambridge Biosciences), Giantin with rabbit anti-Giantin (1:500, Abcam), mouse anti-EEA1 (1:500, BD Transduction Laboratories) and rabbit anti-Transferrin receptor (1:200, Millipore). Actin was detected with Phalloidin-iFluor647 (1:1000, Stratech) as necessary. After labeling, coverslips were washed 3 x PBS and mounted using ProLong Gold Antifade mounting media (Life Technologies). Coverslips were analyzed using a Zeiss Axio Observer Z1 widefield epifluoresence microscope with 100x oil objective, a Leica SP3 confocal microscope or a Leica SP5 confocal microscope, each fitted with a 63x oil objective. For scoring of vesicular EspG in GFP-ARF6 and GFP-ARF6 T44N transfected cells, >100 cells were counted per condition, per biological repeat. For colocalisation analysis, images were deconvolved using the Huygens Professional software package (Scientific Volume Imaging) and Pearson's correlation coefficients calculated using the Coloc2 feature within FIJI[4]. Colocalization analysis was performed on 20 fields of view taken from two independent experiments for each condition. A Pearson's correlation coefficient of 1 indicates perfect colocalization, whilst 0 represents no relationship between the two channels. Confocal microscopy using the Leica SP5 confocal microscope was

preformed at the Facility for Imaging by Light Microscopy (FILM) at Imperial College London.

Immunoblotting

Samples for western blot analysis were boiled prior to separation by SDS-PAGE and transfer to Hybond-P PVDF membranes (GE Healthcare) or Immun-Blot ® PVDF membranes (Bio-Rad). Blotting was carried out according to the manufacturer's instructions. Membranes were blocked in 5% milk/PBS-T (0.02% Tween 20) prior to addition of primary antibodies. Blots were developed with EZ-ECL Chemiluminescence reagent (Geneflow) and visualized with a LAS-3000 imager (Fujifilm).

Co-Immunoprecipitation Assays

HeLa cells expressing GFP-ARF or GFP-Rab constructs were infected with EHEC Δ*espG* + pSA10-EspG:4xHA for five hours. Cells were lysed with lysis buffer (0.5% Triton X-100, 0.5% NP40, 150 mM NaCl, 2 mM EGTA, 2 mM EDTA, 50 mM Tris-HCl pH 7.6) supplemented with cOmplete-mini protease inhibitor tablets (Roche) on ice for 30 minutes. Lysates were centrifuged at 10,000 *x g* for 15 minutes at 4°C before supernatants were harvested. 10% of each supernatant was retained as an input control. Supernatants were pre-cleared with Protein G–coupled Dynabeads (Life Technologies) for 15 minutes at 4°C before incubation with Protein G-coupled dynabeads bound to mouse anti-GFP [9F9.F9] (Abcam). Immunoprecipitation was allowed to proceed for 30 minutes in the case of GFP-ARF constructs and 1 hour in the case of GFP-Rab constructs at 4°C with rotation. For GFP-ARF constructs

beads were washed 1 x lysis buffer, 1 x 0.5% Triton X-100, 1 x 0.05% Tween 20, 1 x 20 mM Tris/200 mM NaCl, 1 x PBS before resuspension in 30 µL 5 x Laemmeli buffer and separation of proteins by SDS-PAGE. For GFP-Rab constructs beads were washed 2 x Lysis buffer, 1 x PBS before resuspension in 5 x Laemmeli buffer and separation by SDS-PAGE. Proteins were analyzed by Western Blot using mouse anti HA-HRP (1:4000, Jackson Immunoresearch) and rabbit anti-GFP (1:2000, Abcam) antibodies.

Statistical Analysis

Results are reported as mean ± Standard Deviation (SD). Statistical analyses were performed with GraphPad PRISM v. 5 using one or two-way analysis of variance (ANOVA) with Bonferroni's post-test. Differences denoted in the text as significant fall below a p-value of 0.05.

1. A. Clements, C.A. Stoneham, R.C. Furniss & G. Frankel (2014) Enterohaemorrhagic Escherichia coli inhibits recycling endosome function and trafficking of surface receptors. *Cell Microbiol* **16**, 1693-705.
2. J. Chun, Z. Shapovalova, S.Y. Dejgaard, J.F. Presley & P. Melancon (2008) Characterization of class I and II ADP-ribosylation factors (Arfs) in live cells: GDP-bound class II Arfs associate with the ER-Golgi intermediate compartment independently of GBF1. *Mol Biol Cell* **19**, 3488-500.
3. G. Montagnac, H. de Forges, E., Smythe, G. Gueudry, M. Romao, J. Salamero & P. Chavrier (2011) Decoupling of activation and effector binding underlies ARF6 priming of fast endocytic recycling. *Curr Biol* **21**, 574-9.
4. J. Schindelin, I. Arganda-Carreras, E. Frise, V. Kaynig, M. Longair, T. Pietzsch, S. Preibisch, C. Rueden, S. Saalfeld, B. Schmid, J.Y. Tinevez, D.J. White, V. Hartenstein, K. Eliceiri, P. Tomancak & A. Cardona (2012) Fiji: an open-source platform for biological-image analysis. *Nat Methods* **9**, 676-82.
